# Artificial Intelligence: The Next Blockbuster Drug in Critical Care?

**DOI:** 10.2478/jccm-2023-0017

**Published:** 2023-05-08

**Authors:** Razvan Azamfirei

**Affiliations:** Department of Anesthesiology and Critical Care Medicine, Johns Hopkins University School of Medicine, Baltimore, MD, USA

The realm of critical care medicine is always waiting for the game-changing innovation – that elusive breakthrough poised to dramatically transform practice and yield remarkable results. Various contenders have come and gone: surfactant therapy, synthetic colloids, drotrecogin alfa [1–3]. While some have been consigned to the annals of history, a select few, such as low-tidal-volume ventilation, have endured [4]. Others re-emerge time and again, hoping that they’ll have an evidence-based role somewhere. The level of excitement is not always correlated with the staying power of the innovation, and sometimes the hype can overshadow the reality.

For the past decade, we witnessed repeated attempts to integrate artificial intelligence techniques into clinical practice [5, 6]. Numerous discussions about the performance of image classification or predictive models have led to minimal real-world progress. Most falter, as their performance declines when they encounter data beyond their training and validation sets [7–9]. Fatalist claims that these models would mean the end of one specialty or another have remained just that – claims [10, 11].

There is a new contender on the block: generative AI systems. These systems are known by various, often imprecise, names such as generative pre-trained transformers, large language models, and neural networks. However, they have become almost synonymous with ChatGPT in the public’s mind. Proponents of Chat-GPT predict that it will revolutionize patient care by expertly summarizing medical records, guiding clinical decisions, and serving as a virtual consultant [12]. Its achievements, such as passing the USMLE Step 1 exam, co-authoring publications, and offering medical advice, lend credence to this idea [13–15].

Large language models (LLMs) seem to possess extraordinary natural language processing abilities. They appear to understand, summarize, and generate text independently. Consequently, LLMs could swiftly synthesize and summarize copious amounts of medical literature, assimilate data from various patient chart sources, and apply the latest guidelines to support decision-making—a computer boasting perfect memory that seemingly “comprehends” clinical context and pathology. However, we must ask ourselves: is this understanding genuine or are we dwelling in a Potemkin village?

History cautions us that enthusiasm is rarely warranted in critical care. Searching for understanding behind the painted walls, we discover something else. Answers stemming not from genuine understanding, but from predictions [16]. What we find is a very sophisticated model that generates content by predicting which words are likely to appear in similar contexts. While LLMs excel when processing familiar concepts, they falter when confronted with abstract or unusual scenarios [17].

The distinction between a physician and a layperson lies not in textbooks but in experience and comprehension of disease processes. Although it is improbable that AI will practice independently, we are likely to see systems employing a human-in-the-loop strategy. By blending AI’s computational prowess with human clinicians’ nuanced understanding, AI can function as a decision aid rather than a decision maker. Some practitioners have already encountered this approach in the sepsis prediction model used by major EHRs [7]. However, they may have also observed its subpar performance or its tendency to “cheat”, relying on clinicians initiating sepsis treatment to predict sepsis presence [18]. It is not just an example of AI gone awry, but it exemplifies the ethical and practical challenges arising from AI algorithms’ opacity[19]. As models grow more complex and their decision-making processes become increasingly obscure, concerns about our ability to comprehend their recommendations arise. Sometimes, there is no human-friendly justification. Mistrust in the system undermines its role. But overreliance, or blindly following the system takes the human out of the loop.

We must note AI’s potential to exacerbate existing disparities in healthcare. Bias in AI algorithms can originate from multiple sources, including biased training data, flawed model assumptions, and the impact of historical practices [9, 20]. This may perpetuate systemic inequalities, particularly for vulnerable or marginalized populations, masking the bias beneath a veneer of scientific impartiality, or hiding it behind an opaque algorithm [21].

This is not to imply that these systems lack tremendous potential. When implemented appropriately, they could offer substantial benefits or aid in streamlining tedious tasks. If this enables clinicians to devote more time to direct patient care, it is a victory. After all, true care and empathy cannot be programmed.

I share the enthusiasm towards AI and my intent is not to diminish its potential role. Rather, I ask that we approach this technology with the same skepticism and apply the same degree of scrutiny as we do when evaluating other medical interventions. The future of critical care will not hinge on a single, revolutionary innovation, but on incremental steps, each edging us closer to our goal: providing the best possible care. I hope that AI is one of those steps, but we should remember: you cannot program empathy and genuine care.

## References

[j_jccm-2023-0017_ref_001] Lewis JF, Brackenbury A. (2003). Role of exogenous surfactant in acute lung injury. Crit Care Med.

[j_jccm-2023-0017_ref_002] Zarychanski R, Abou-Setta AM, Turgeon AF (2013). Association of hydroxyethyl starch administration with mortality and acute kidney injury in critically ill patients requiring volume resuscitation: a systematic review and meta-analysis. JAMA.

[j_jccm-2023-0017_ref_003] Savel RH, Munro CL. (2012). Evidence-based backlash: the tale of drotrecogin alfa. Am J Crit Care.

[j_jccm-2023-0017_ref_004] Brower RG, Matthay MA, Acute Respiratory Distress Syndrome Network (2000). Ventilation with lower tidal volumes as compared with traditional tidal volumes for acute lung injury and the acute respiratory distress syndrome. N Engl J Med.

[j_jccm-2023-0017_ref_005] Feretzakis G, Karlis G, Loupelis E (2022). Using Machine Learning Techniques to Predict Hospital Admission at the Emergency Department. J Crit Care Med (Targu Mures).

[j_jccm-2023-0017_ref_006] Lundberg SM, Nair B, Vavilala MS (2018). Explainable machine-learning predictions for the prevention of hypoxaemia during surgery. Nat Biomed Eng.

[j_jccm-2023-0017_ref_007] Lyons PG, Hofford MR, Yu SC (2023). Factors Associated With Variability in the Performance of a Proprietary Sepsis Prediction Model Across 9 Networked Hospitals in the US. JAMA Intern Med.

[j_jccm-2023-0017_ref_008] Vela D, Sharp A, Zhang R (2022). Temporal quality degradation in AI models. Sci Rep.

[j_jccm-2023-0017_ref_009] Varoquaux G, Cheplygina V. (2022). Machine learning for medical imaging: methodological failures and recommendations for the future. NPJ Digit Med.

[j_jccm-2023-0017_ref_010] Mazurowski MA. (2019). Artificial Intelligence May Cause a Significant Disruption to the Radiology Workforce. J Am Coll Radiol.

[j_jccm-2023-0017_ref_011] Krittanawong C. (2018). The rise of artificial intelligence and the uncertain future for physicians. Eur J Intern Med.

[j_jccm-2023-0017_ref_012] Lee P, Bubeck S, Petro J. (2023). Benefits, Limits, and Risks of GPT-4 as an AI Chatbot for Medicine. N Engl J Med.

[j_jccm-2023-0017_ref_013] Kung TH, Cheatham M, Medenilla A (2023). Performance of ChatGPT on USMLE: Potential for AI-assisted medical education using large language models. PLOS Digit Health.

[j_jccm-2023-0017_ref_014] Salvagno M, Taccone FS, Gerli AG. (2023). Can artificial intelligence help for scientific writing?. Crit Care.

[j_jccm-2023-0017_ref_015] Ayers JW, Poliak A, Dredze M (2023). Comparing Physician and Artificial Intelligence Chatbot Responses to Patient Questions Posted to a Public Social Media Forum. JAMA Intern Med.

[j_jccm-2023-0017_ref_016] Vaswani A, Shazeer N, Parmar N, Guyon I, Luxburg UV, Bengio S, Wallach H, Fergus R, Vishwanathan S (2017). Attention is All you Need.

[j_jccm-2023-0017_ref_017] Liu H, Ning R, Teng Z (2023). Evaluating the Logical Reasoning Ability of ChatGPT and GPT-4. arXiv preprint arXiv:230403439.

[j_jccm-2023-0017_ref_018] Wong A, Otles E, Donnelly JP (2021). External Validation of a Widely Implemented Proprietary Sepsis Prediction Model in Hospitalized Patients. JAMA Intern Med.

[j_jccm-2023-0017_ref_019] Smith H. (2020). Clinical AI: opacity, accountability, responsibility and liability. Ai & Society.

[j_jccm-2023-0017_ref_020] Daneshjou R, Smith MP, Sun MD, Rotemberg V, Zou J. (2021). Lack of Transparency and Potential Bias in Artificial Intelligence Data Sets and Algorithms: A Scoping Review. JAMA Dermatol.

[j_jccm-2023-0017_ref_021] Noseworthy PA, Attia ZI, Brewer LC (2020). Assessing and Mitigating Bias in Medical Artificial Intelligence: The Effects of Race and Ethnicity on a Deep Learning Model for ECG Analysis. Circ Arrhythm Electrophysiol.

